# Medical Colleges and Graduates in the United States

**Published:** 1871-12

**Authors:** 


### Medical Colleges and Graduates in the United States.

We are informed by private correspondence that Dr. Toner's' statistics for the Department of the Interior, show in the United States.

MEDICAL TEACHING BODIES.

| | |
|---|---|
| Regular | 60 |
| Pharmacy | 16 |
| Dental | 8 |
| Homœopathic | 8 |
| Eclectic | 6 |
| Botanic | 2 |

Forty-eight of the colleges have furnished statistics of 1870 which show 4,989 Matriculants; 1,500 Graduates; 77 *Ad Eundem*, and 15 honorary degrees.

